# Detection of calcium pyrophosphate dihydrate crystals in knee meniscus by dual-energy computed tomography

**DOI:** 10.1186/s13018-018-0787-0

**Published:** 2018-04-05

**Authors:** Hidenori Tanikawa, Ryo Ogawa, Kazunari Okuma, Kengo Harato, Yasuo Niki, Shu Kobayashi, Takeo Nagura

**Affiliations:** 1 Department of Orthopaedic Surgery, Saiseikai Yokohamashi Tobu Hospital, 3-6-1 Shimosueyoshi, Tsurumi, Yokohama, Kanagawa Japan; 2 Department of Orthopaedic Surgery, Keio University School of Medicine, 35 Shinamomachi, Shinjyuku, Tokyo, Japan; 3 Department of Orthopaedic Surgery, Saitama City Hospital, 2460 Minuma, Midoriku, Saitamashi, Saitama, Japan; 4 Department of Clinical Biomechanics, Keio University School of Medicine, 35 Shinamomachi, Shinjyuku, Tokyo, Japan

**Keywords:** Dual-energy computed tomography, Calcium pyrophosphate, Knee joint meniscus, Pseudogout

## Abstract

**Background:**

Calcium pyrophosphate dihydrate (CPPD) crystals are commonly observed in osteoarthritic joints. The aim of our study was to investigate the efficacy of a dual-energy computed tomography (DECT) for detecting CPPD crystals in knee meniscus.

**Methods:**

Twenty-six patients undergoing primary total knee arthroplasty were included in the study. Radiographs of knee joint and synovial fluid specimens were analyzed for the presence of CPPD crystals. Meniscus extracted during surgery was scanned using DECT. Sensitivity and specificity of DECT and radiograph for detecting CPPD crystals were calculated against a reference standard (polarizing light microscopy of synovial fluid aspirate). Meniscus in which CPPD crystals were suspected with DECT was further examined to confirm the crystals using a polarized microscopy.

**Results:**

CPPD crystals in synovial fluid were observed in 9 (36%) patients. The sensitivity and specificity of DECT in the detection of CPPD crystals, against microscopic identification, were 77.8 and 93.8%, respectively. The sensitivity and specificity of conventional radiography in the detection of CPPD crystals were 44.4 and 100%, respectively. DECT was able to detect the area where CPPD crystals were deposited in the meniscus.

**Conclusion:**

DECT provides good diagnostic sensitivity and specificity for detection of CPPD crystals in knee meniscus as well as spatial information about CPPD crystals. DECT is currently a research tool, but we believe that DECT can be a useful instrument to diagnose CPPD deposition disease, especially for the regions where aspiration is difficult to be performed such as pubic symphysis, atlantoaxial joint, interphalangeal joint.

## Background

Calcium pyrophosphate dihydrate (CPPD) deposition disease sometimes causes acute inflammatory mono-articular arthritis known as pseudogout. CPPD deposition disease occurs mainly in the elderly population, and the crystals are observed in osteoarthritic joints [1]. CPPD crystals around the knee joint can be observed in all articular and peri-articular tissues, such as cartilage, synovium, and ligaments; however, it is most commonly found in hyaline articular cartilage and meniscal fibrocartilage [2]. According to the population-based studies of CPPD deposition disease prevalence, 13–30% of knee specimens harvested at the time of surgery for a diagnosis of knee osteoarthritis contained CPPD crystals [3–5]. The pathogenic role of calcium contain crystals, including CPPD and basic calcium phosphate, is still unclear and controversial [6, 7]. However, growing clinical and experimental evidence indicates that these crystals may induce microcrystal stress on synoviocytes and chondrocytes, leading to exacerbation of osteoarthritis [8].

Presently, two clinical tests are used to detect CPPD crystals. The first and most specific test is visualization of weakly birefringent rhomboid CPPD crystals in synovial fluid aspirated from the affected joint. However, reliance on morphologic crystal identification is risky as crystals may not be seen in a single synovial sample and the smallest crystals can be easily missed [9]. Observation of the linear calcification on radiograph is often used to diagnose CPPD deposition disease, but it is not highly sensitive [10]. Methodology advances, such as the utility of high-resolution ultrasound, have attracted recent attention, yet chondrocalcinosis detected by plain radiography remains the primary screening approach, with advantages including universal availability, being technically undemanding and giving panoramic imaging of the joint [1, 11].

Although dual-energy computed tomography (DECT) was first conceived in 1976, it has not been used widely for clinical indications [12]. The principle of dual-energy imaging is based on physical properties of the examined material in terms of how the material interacts with electromagnetic radiation [12, 13]. In general, x-rays having a different energy of the emitted photons are absorbed differently and therefore differ in attenuation in CT scans acquired with high or low tube voltage. Thus, a dual-energy gradient can be calculated, which characterizes this differing x-ray interaction and is relatively specific for a given material [14–17]. Recent studies reported that DECT was able to distinguish CPPD crystals from uric acid crystals and was useful to diagnose CPPD deposition disease, especially when crystal examination was not available or radiography was not easily interpretable [17, 18].

The aim of our study was to compare the efficacy of the DECT for detecting CPPD crystals in knee meniscus to that of a conventional radiography. Our hypothesis is that a DECT has better sensitivity and specificity in detecting CPPD crystals than a conventional radiography.

## Methods

This was a prospective study conducted in one center. We enrolled 26 patients, 3 males, and 23 females, undergoing primary total knee arthroplasty (TKA) due to severe osteoarthritis (OA). The OA grading (Kellgren-Lawrence grading) was grade 3 for 11 patients and grade 4 for 15 patients. Exclusion criteria included patients with rheumatoid arthritis or osteonecrosis, bilateral TKA. This study was conducted according to the Declaration of Helsinki and local regulations. The institutional ethics committee approved the study and informed consent was obtained from all patients.

Preoperatively, radiographs of the knees (standard anteroposterior and lateral views) were analyzed for the presence of calcification by a radiologist. The grade of knee OA radiographic severity was classified according to the criteria of Kellgren and Lawrence [19]. The joint fluid was aspirated just before starting the operation by an orthopedic surgeon and was analyzed using a polarized light microscopy (BX50, Olympus Optical, Tokyo, Japan). The meniscus extracted during surgery was preserved in saline solution and was scanned with DECT system (Somatom Definition Flash, Siemens Healthcare, Forchheim, Germany). The DECT images were evaluated by an expert radiologist who was blinded to radiographic findings for the presence of CPPD crystals. Finally, the meniscus with crystals was fixed in 10% neutral-buffered formalin for 48 h, embedded in paraffin, and then sectioned at 5 μm at the point where CPPD deposition was observed by DECT. Sections were stained with hematoxylin and eosin for evaluation for the presence of CPPD crystals using a polarized microscopy.

### Scanning technique

**Fig. 1:**
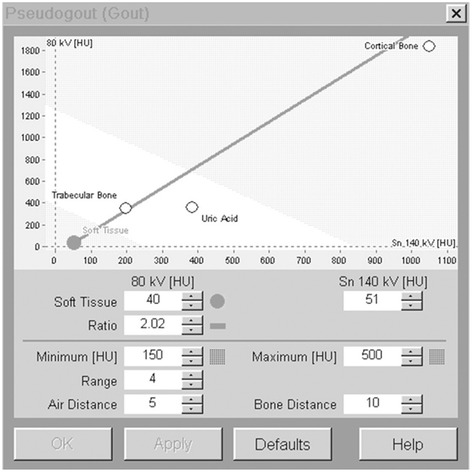
Screenshot of the dual-energy CT post-processing software showing the settings for generation of calcium pyrophosphate dihydrate crystals images in the knee joint

CT examinations were performed by using a dual-source CT scanner with tube potentials of 80 and 140 kV. To increase the separation of the two x-ray spectra, the DECT is equipped with a tin filter in the high energy beam [20]. The dual-source scanner allows simultaneous acquisition at two different energy levels and creation of two different data sets that are loaded into the post-processing software (Syngo Dual Energy, Siemens Healthcare, Forchheim, Germany). In CT value plots, with the *y*-axis representing the attenuation values of the lower kilovoltage tube (80 kv), and the *x*-axis representing the attenuation values of the higher kilovoltage tube (140 kv), base materials should appear as a straight line. In addition to the slope of the separation line, maximum and minimum values can be defined for soft tissue. The scanning parameters used for this particular application are as follows: 140 kV and 85 mAs for tube A, and 80 kV and 157 mAs for tube B. For both tubes, collimation of 0.6 mm is reconstructed to 0.1 mm transverse thick slices obtained at 80 and 140 kV. The base material “soft tissue” was chosen to be at 80 kV (40 HU) and at 140 kV (51 HU). Best results were obtained by setting the parameter ratio to 2.02. The range of values for the calculation was set between 150 and 500 (Fig. 1). We expressed standard descriptive statistics, including proportions for microscopic findings and means and SDs for demographic data. SPSS version 15.0 (SPSS, Chicago, IL) was used for statistical analysis. The McNemar test was employed to determine if there was a difference between DECT and conventional radiography in detecting the CPPD crystals, with an alpha level set at 0.05.

## Results

**Fig. 2:**
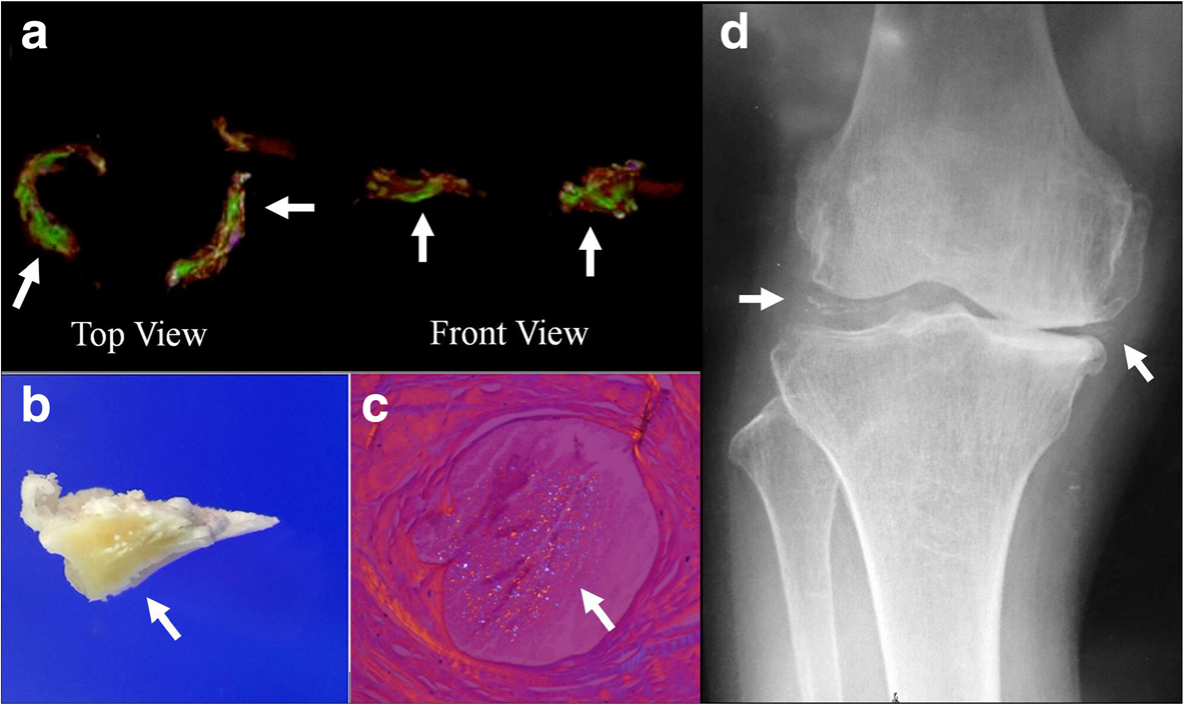
**a** Dual-energy CT image of the meniscus reconstructed as a three-dimensional model (top view and front view), in which CPPD crystals (arrows) are colored in green. **b** CPPD crystals (arrow) are grossly observed in the meniscus. **c** Pathological analysis of a sample of the meniscus, confirming the presence of CPPD crystals at the site indicated by dual-energy CT. Rhomboid-shaped and rectangular-shaped CPPD crystals (arrow) with a weakly positive birefringence pattern are observed. **d** A Radiograph of the knee joint with liner calcifications (arrows) in the medial and lateral meniscus

CPPD crystals in synovial fluid were observed in 9 (36%) patients. The sensitivity and specificity of DECT in the detection of CPPD crystals against microscopic identification as the gold standard were 77.8 and 93.7%, respectively (Table 1). The sensitivity and specificity of conventional radiography in the detection of CPPD crystals were 44.4 and 100.0%, respectively (Table 2). There was no significant difference between the sensitivity of DECT and that of conventional radiography (*p* = 0.248). Concerning the pathological examination, CPPD crystals were observed in every meniscus in which CPPD crystals were indicated by DECT. Furthermore, the location of the CPPD crystal by microscopic analysis was consistent with the colored area of DECT (Fig. 2).

**Table 1:** Relationship between the DECT findings of meniscal calcification and microscopic identification of CPPD crystals

	Microscopic identification of CPPD crystals		
	Present	Absent	Total
DECT findings			
Present	7 (28%)	1 (4%)	8 (32%)
Absent	2 (8%)	15 (60%)	17 (68%)
Total	9 (36%)	16 (64%)	25 (100%)

Values are the number or number (percentage)

*DECT* dual-energy computed tomography, *CPPD* calcium pyrophosphate dihydrate

**Table 2:** Relationship between the conventional radiography findings of meniscal calcification and microscopic identification of CPPD crystals

	Microscopic identification of CPPD crystals		
	Present	Absent	Total
Radiography			
Present	4 (16%)	0 (0%)	4 (16%)
Absent	5 (20%)	16 (64%)	21 (84%)
Total	9 (36%)	16 (64%)	25 (100%)

Values are the number or number (percentage)

*CPPD* calcium pyrophosphate dihydrate

## Discussion

Our principal finding was that DECT had a higher sensitivity than conventional radiography for the detection of CPPD crystals (77.8 versus 44.4%), whereas DECT had a similar specificity to conventional radiography (93.7 versus 100%). Although the difference in the sensitivities did not reach significance (*p* = 0.248) possibly due to the small number of subjects, to our knowledge, this is the first study which researched the sensitivity and specificity of DECT for CPPD crystals. Previous studies have found that DECT was useful to diagnose CPPD deposition disease in knee joint or distal radioulnar joint, but did not researched the sensitivity or specificity of the DECT [19, 21]. The good sensitivity of DECT have been reported in the previous studies assessing the usability of DECT for gout, which documented that the sensitivity of DECT was over 90% [22, 23]. Compared to these reports, our result showed a relatively low sensitivity of DECT for CPPD crystals (77.8%). This discrepancy may be due to the difference of crystal components, or due to our study design. The weakness of this study was that we did not analyze the patients’ knee joint but analyzed only the extracted meniscus. Therefore, if the CPPD crystals were deposited only at other soft tissues such as the anterior cruciate ligament or articular cartilage, DECT could not detect the crystals. Two patients had a negative test result in DECT but also had a positive test result in joint fluid analysis. Besides the study design, this false-positive result may be due to the quantity of the CPPD crystals that could be below the detection limit of DECT. A previous phantom study showed that single source DECT allowed reliable detection of CPPD crystals at concentrations of 6.25% or higher [17]. In the current study DECT detected CPPD crystals in a patient with severe osteoarthritis, although microscopic analysis did not found CPPD crystals in her joint fluid. Similar results were documented in previous studies about DECT in gout [22, 24]. DECT demonstrated evidence of monosodium urate deposition in seven cases among the 41 patients with severe osteoarthritis who had negative results with synovial fluid analysis [23]. It is unclear whether these deposits in DECT represent an imaging artifact or indicate subclinical CPPD deposition which occur only inside the meniscus.

One of the clinical advantages of DECT is that it is able to differentiate articular monosodium urate from CPPD crystals [18]. Previous reports found that DECT is a highly accurate noninvasive method for detecting different urinary stone types [25]. In the same way, DECT can be used to diagnose CPPD deposition disease and gout without joint aspiration [18, 26]. Two case reports documented that DECT was useful to diagnose CPPD deposition disease in knee and wrist joints [19, 21]. Furthermore, DECT can examine all the regions where aspiration is difficult to be performed, such as pubic symphysis, atlantoaxial joint, interphalangeal joint, wrist joint and so on. Although the expense, availability, and clinical accuracy of this technology will need to be addressed before it becomes a useful clinical tool, DECT is the only imaging modality which can distinguish monosodium urate crystals from CPPD crystals and can examine all joints in a body.

Besides conventional radiography, several studies suggest that ultrasound be more sensitive for the detection of chondrocalcinosis than conventional radiography [11, 27]. The previous study showed that meniscal chondrocalcinosis was detected by ultrasound in 90% of the patients with CPPD and the specificity of ultrasound was 100% against the detection of synovial fluid CPPD crystals [27]. Although the ultrasound technique has good sensitivity and good specificity and needs no radiation exposure, the technique is very operator-dependent [11]. In addition, for patients with late stage osteoarthritis who have marked osteophytosis at the medial and lateral compartment of the knee, it is difficult to obtain sufficiently high-quality images to permit the correct evaluation.

One disadvantage should be noted regarding DECT. Unlike ultrasound sonography or radiography, the DECT involves much radiation. DECT exposure level is almost the same as conventional CT scan that has a dose of approximately 0.16 mSv for knee joint examination [28]. Although there is no evidence that an effective dose of less than 10 mSv causes harmful medical effects, it is desirable to lower the effective dose as much as possible [29].

Our study has several limitations that need to be considered when interpreting our data. The study lacks the evaluation of DECT in situ. From a patient-care perspective, DECT should be performed to the patient’s knee joint instead of the extracted meniscus to diagnose any potential problems in the knee joint. To clarify the real strength of DECT to diagnose CPPD deposition disease, future research assessing a knee joint in situ is necessary. Also, the small number of the patients was not enough to make an exact assessment of sensitivity and specificity of DECT in CPPD crystals.

## Conclusions

In conclusion, DECT demonstrate good sensitivity and specificity in the detection of CPPD crystals in meniscus. Additionally DECT provides spatial information for CPPD deposition. DECT is currently a research tool, but we believe that DECT can be a useful tool to diagnose CPPD deposition disease especially for the regions where aspiration is difficult to be performed such as pubic symphysis, atlantoaxial joint, and interphalangeal joint.

## Abbreviations

CPPD: Calcium pyrophosphate dihydrate
CT: Computed tomography
DECT: Dual-energy computed tomography
OA: Osteoarthritis
TKA: Total knee arthroplasty
